# Insulin therapy and colorectal cancer risk among type 2 diabetes mellitus patients: a systemic review and meta-analysis

**DOI:** 10.1186/1746-1596-9-91

**Published:** 2014-05-12

**Authors:** Shinan Yin, Hua Bai, Danqing Jing

**Affiliations:** 1 Department of Endocrinology, First affiliated hospital of General hospital of PLA, Beijing 100048, China

## Abstract

**Background:**

Insulin is widely used in patients with type 2 diabetes mellitus (T2DM). More attention was focused on its higher risk of colorectal cancer (CRC). This meta-analysis examined the relationship between levels of insulin use and the risk of CRC.

**Methods:**

A meta-analysis using data from 12 published epidemiologic studies (7 case–control, and 5 cohort studies) published before Jan. 2014 was done to examine the association between insulin use and CRC. Random effects analyses were done to calculate relative risk (RR) and 95% confidence intervals (CI). Heterogeneity among studies was measured by the χ^2^ and I^2^ statistic.

**Results:**

Overall, the risk of CRC was significantly associated with insulin use to a random-effects model (RR, 1.69; 95% CI, 1.25 -2.27). When subgroup analyses were conducted according to the study types, no associations were detected in cohort group (RR, 1.25; 95% CI, 0.95-1.65; I^2^, 75.7%); however significant association was detected in case–control group (RR, 2.15; 95% CI, 1.41-3.26; I^2^, 89.1%).

**Conclusions:**

A significant harmful effect of insulin, observed mainly among case–control studies, may result from study design differences and amount of included studies. Although these results suggest a harmful effect of insulin use for CRC risk, additional large studies are warranted to support these preliminary evidences.

**Virtual Slides:**

The virtual slide(s) for this article can be found here: http://www.diagnosticpathology.diagnomx.eu/vs/2194715731194123.

## Background

Colorectal cancer (CRC) is now the second leading cause of cancer-related death in the developed countries [1]. Previous epidemiological studies showed that smoking, alcohol consumption, and obesity are risk factors for colorectal cancer [2]. It was proposed that the effects of dietary and lifestyle risk factors on CRC may be mediated through hyperinsulinemia. Type 2 diabetes mellitus (T2DM), a condition associated with hyperinsulinemia, is associated with an increased risk of mortality from a range of solid tumors, including cancers of the colon, breast and pancreas [3,4]. Similar associations have been noted with central obesity and other conditions associated with increased levels of circulating insulin [5,6]. These observations have given rise to the hypothesis that growth of these tumors, which are characterized by abnormal expression and function of the insulin–IGF-1 series of receptors, is promoted by the trophic action of insulin interacting with these receptors [7,8]. Some results showed that insulin use would increase cancer risk, while others suggested that insulin did not play a role in cancer development. Furthermore, in an animal model, exogenous insulin injection stimulates the growth of colorectal cancer precursors [9]. In clinical studies, high circulating insulin levels are independently associated with increased colorectal cancer risk. Patients with T2DM begin to require insulin therapy when there is significant decline in endogenous insulin production. However, hyperinsulinemia is actually augmented during this phase by exogenous insulin because of the inefficiency of exogenous insulin. Based on these observations, we hypothesized that chronic exogenous insulin therapy among patients with T2DM may promote colorectal cancer development.

Meta-analysis is a useful statistical tool to pool the relevant studies together and gain a more powerful conclusion [10]. The meta-analysis was also used in the search for potential causes of CRC. For instance, Boyle T et al. searched MEDLINE and EMBASE for English-language cohort and case–control studies that examined associations between physical activity and the risks of CRC. Through combining twenty-one studies, the results of the systematic review and meta-analysis suggest that physical activity is associated with a reduced risk of CRC, and that the magnitude of the association does not differ by subsite [11]. Nowadays, the association between insulin use and risk of CRC is still unclear and a meta-analysis would provide more powerful evidence. Given these reasons, the aim of the current meta-analysis and systematic review was to quantitatively evaluate findings from observational studies on the insulin use and the incidence of CRC in patients with T2DM.

## Methods

### Search strategy and inclusion criteria

This meta-analysis was conducted according to the PRISMA guidelines [12]. We searched PubMed and EMBASE to retrieve related studies published before Jan, 2014 and Medical Subject Heading (MeSH) keywords “colorectal” and “cancer”, “carcinoma”, “neoplasia”, “tumor” in combine with “insulin”, “antihyperglycemic”. The citations of related articles were detected for additional publications. When several reports from the same study were published, only the most recent or informative one was included in this meta-analysis. The language was restricted to only English. Contacting to the corresponding authors of retrieved articles was conducted when additional information was needed.

The articles would be considered eligible if the studies met the inclusion criteria: [1] evaluate the association between insulin use and risk of CRC; [2] adopt case–control or cohort study design; [3] provides the odds ratios (ORs) or relative risks (RRs) with confidence intervals (CI), standard errors or sufficient data to calculate them.

### Data extraction and assessment of study quality

Two reviewers (S.Y and D.J) extracted the data from each study independently and checked again after the first extraction. Any disagreements about data extraction were discussed by two reviewers and resolved finally. The data extracted from each study contained: name of first author, study design, study period, country, numbers of subjects (cases, controls, or total), adjustments of the related factors, exposure definition and OR/RR value with 95% CI.

Considering that only observational studies were included in this current meta-analysis, the methodological qualities of the included studies were assessed using the Newcastle-Ottawa scale (NOS) [13]. The NOS was developed for the study quality assessment of the observational studies. It assessed the selection, comparability and exposure or outcome of a case–control or cohort study. The maximum of NOS was 9 stars for a study and the study with over 6 stars was regarded as relatively high quality. The quality scale was assessed by two reviewers (S.Y and D.J) and disagreements were resolved through discussion with the third reviewer (H.B).

### Statistical methods for the meta-analysis

Expected heterogenicity of the methodology, data source and so on existed in the included studies. Accordingly, random-effect methods were used to pool the association between insulin use and risk of CRC for all analyses [14]. The effect was combined under the assumption that ORs were accurate approximations of RRs. When both adjusted and unadjusted data were available, the adjusted data (adjusted ORs or RRs with 95% CI) were used to compare the exposed and unexposed of aspirin use. If only stratified results (e.g., by CRC subtypes) were provided, fixed-effect methods were obtained to summarize the results into a single parameter for each study. Meanwhile, subgroup analyses were carried out by study designs and sites. Statistical heterogeneity across studies was evaluated by both the χ^2^ and *I*^*2*^ tests. If P < 0.1 and *I*^*2*^ > 50%, the interstudy heterogeneity was regarded statistically significant. When the heterogeneity couldn’t be ignored, subgroup analyses and meta-regression would be conducted to explore the source of heterogeneity.

Sensitivity analyses were conducted to detect the robustness of the outcome. After excluding the studies with lower NOS scale, the studies with higher quality were included in the sensitivity analyses. Meanwhile, sensitivity analyses were conducted by changing the fixed-effect methods to random-effect methods. Potential publication bias was assessed via both visually evaluating a funnel plot and the Egger test [15,16]. All the analyses were conducted using the Stata software package (version 12.0; Stata Corp., College Station, TX).

## Results

### Identification and selection of studies

**Figure 1:**
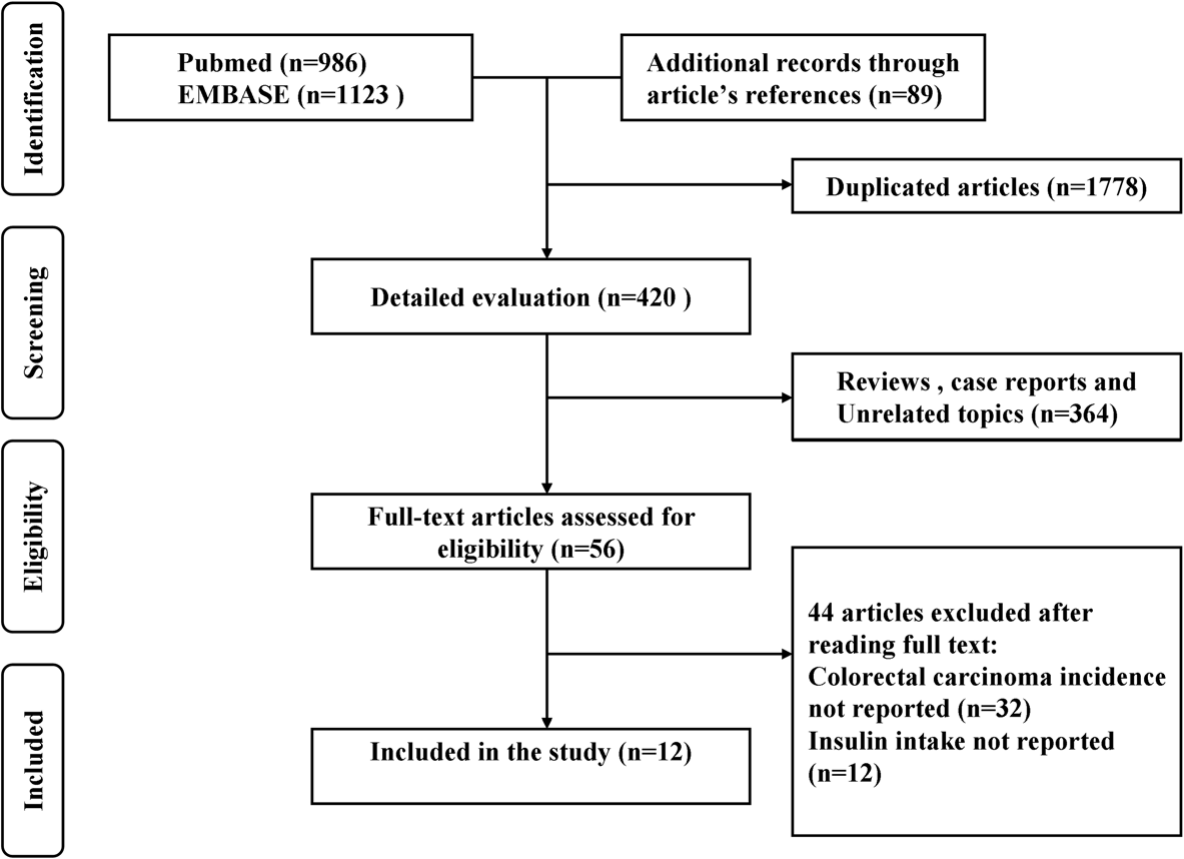
**Flow chart of the literature search.** The literature search was conducted in Medline and EMBASE. The reference lists of the relevant studies were reviewed as well.

The initial 2109 articles (986 from PubMed and 1123 from Emabse) were identified. Besides, 89 articles were included from reviewing the reference lists of the related articles. After 1778 duplicates and 364 unrelated articles were excluded, 56 full-text articles were assessed for eligibility. Among the 56 articles, 32 articles that didn’t report the incidence of CRC and 12 articles that didn’t report insulin intake were excluded. In final, 12 studies were included in this meta-analysis [17-28]. Figure 1 provides a flow of search results.

### Study characteristics and quality

A total of 491384 individuals were included in this current meta-analysis. The characteristics of these included studies were demonstrated in Table 1. Among the 12 included studies, 7 studies were case–control studies and 5 studies were cohorts. Geographic distribution of all included studies was 5 in Americas, 3 in European and 4 in Asia. To evaluate the methodological qualities of the included studies, the NOS method was used in current meta-analysis. The NOS quality assessment score of the most studies was > 6 (mean: 6.83; standard deviation: 1.23) and only one study got a less than 6 stars because the shortages in data source or methodological designs.

**Table 1:** Study characteristics of included cohort and case–control studies on insulin use and CRC risk

**Study, Year**	**Study period**	**Study design**	**Country**	**No. of participants**	**No. of cases**	**Duration of insulin use**	**Gender**	**Confounders for adjustment**
Campbell 2010 [17]	1992-2007	Cohort	USA	184,194	2,474	≥4 years	M/F	1, 3, 4, 5, 6, 7, 8, 20
Carstens 2012 [18]	1995-2009	Cohort	Denmark	22,826	320	NR	M/F	1, 9, 10
Chang 2012 [19]	2000-2007	C-C	China	108,920	468	≥2 years	M/F	11, 12, 13, 14, 15, 16, 17
Chung 2008 [20]	2003-2006	C-C	Korea	325	100	NR	M/F	1, 2
Currie 2009 [21]	2000–2009	Cohort	UK	62,809	292	NR	M/F	1, 2, 18, 19
Gu 2013 [22]	2001-2010	Cohort	China	8,774	31	NR	M/F	1,2, 11, 18,
Hsieh 2012 [23]	2000-2008	C-C	China	61,777	1,739	NR	M/F	1, 2
Koro 2007 [24]	1997–2004	C-C	USA	2,435	408	NR	M/F	1, 2, 9, 10
Onitilo 2013 [25]	1995-2009	Cohort	USA	9,486	106	NR	M/F	1, 3, 11, 12
Vinikoor 2009 [26]	1996-2006	C-C	USA	3,752	1,688	≥1 years	M/F	1,2, 5, 7, 20, 21
Wong 2012 [27]	1998-2007	C-C	USA	1,168	196	≥3 years	M/F	1, 2
Yang 2004 [28]	1987-2002	C-C	UK	24,918	125	≥5 years	M/F	2, 3, 5, 8, 11, 18

C-C: Case–control study; M: male; F: female.

The adjusteted factors are: (1) age, (2) gender, (3) body mass index, (4) physical activity, (5) nonsteroidal anti-inflammatory drug use, (6) alcohol use, (7) family history of colorectal cancer, (8) endoscopy history, (9) current date of follow-up; (10) date of birth (11) use of sulfonylurea, glinides, metformin, thiazolidinediones, α-glucosidase inhibitors (11) chronic liver disease, (12) nephropathy, (13) statins, β-blocker, calcium channel blockers, (14) cerebrovascular disease, (15) angiotensin-converting enzyme inhibitors, (16) chronic kidney disease, (17) aspirin, (18) smoking status, (19) diagnosis of a previous cancer; (20) education; (21), calcium intake.

### Insulin use and risk of CRC

**Figure 2:**
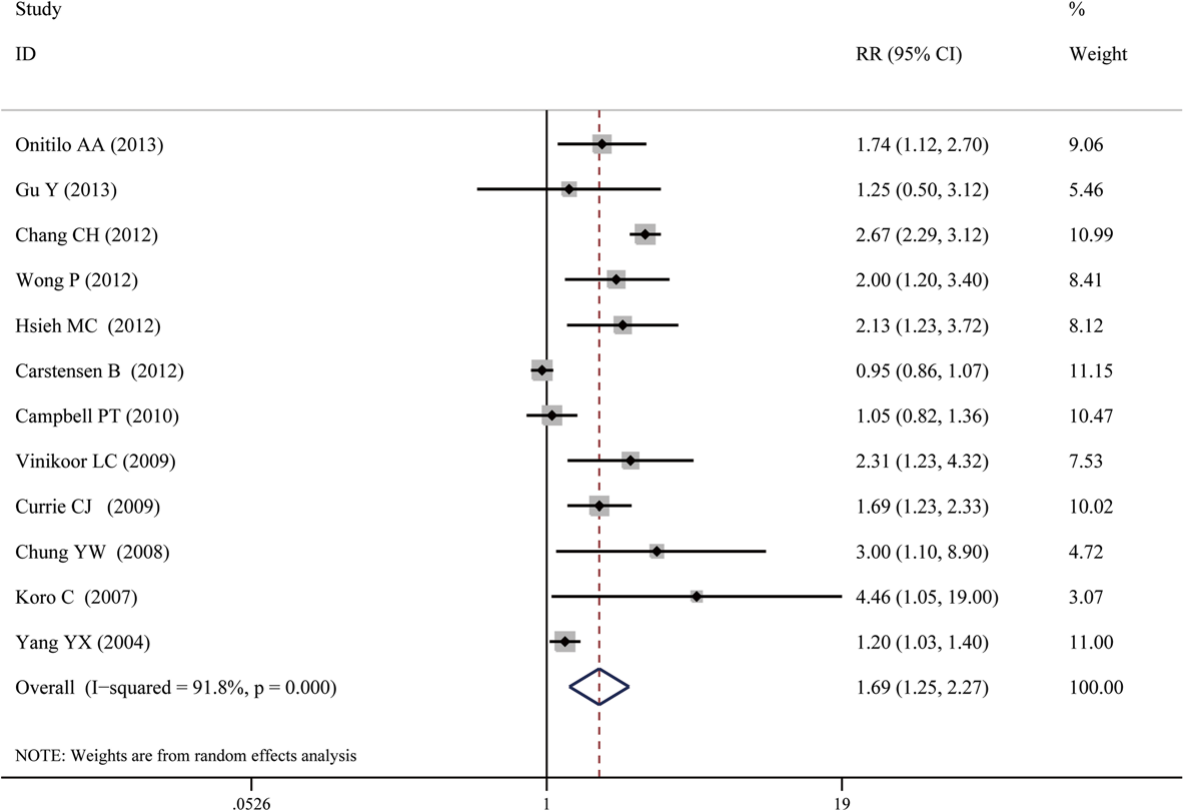
**Forest plot of the association between insulin use and risk of CRC.** The size of the shaded square is proportional to the percent weight of each study. The horizontal lines represent 95% CIs. The diamond data markers indicate the pooled ORs. A random-effect model was obtained.

Figure 2 shows the effect of insulin use and risk of CRC. In a random-effects meta-analysis, the use of insulin was associated with increased risk of CRC (RR, 1.69; 95% CI, 1.25 -2.27; I^2^, 91.8%). Table 2 displayed the effects of insulin use and CRC risk in subgroup analysis by study designs and sites. When subgroup analyses were conducted according to the study types, no associations were detected in cohort group (RR, 1.25; 95% CI, 0.95-1.65; I^2^, 75.7%); however significant association was detected in case–control group (RR, 2.15; 95% CI, 1.41-3.26; I^2^, 89.1%).The significant association between insulin use and CRC risk was detected in the studies in America (RR, 1.73; 95% CI, 1.15-2.60; I^2^ = 67.8%) and Asia (RR, 2.55; 95% CI, 2.14-3.04; I^2^ = 3.9%). No significant from the studies from Europe was found (RR, 1.20; 95% CI, 0.92-1.57; I^2^ = 85.8%). When the gender was considered, however, no association was detected in neither male nor female group. However this result should be considered in caution considering that only 3 studies were included in each group (Table 3).

**Table 2:** **Quality assessment of included studies **^**1**^

**Author**	**Quality assessment criteria--Case–control**			
	**Selection**	**Comparability**	**Outcome/Exposure**	**Overall quality**
Chang et al. 2012 [19]	***	**	***	8
Chung et al. 2008 [20]	***	*	**	6
Hsieh et al. 2012 [23]	***	**	***	8
Koro et al. 2007 [24]	***	*	**	6
Vinikoor et al. 2009 [26]	**	*	**	5
Wong et al. 2012 [27]	***	**	**	7
Yang et al. 2004 [28]	***	**	**	7
**Author**	**Quality assessment criteria—Cohort**			
	**Selection**	**Comparability**	**Outcome/Exposure**	**Overall quality**
Campbell et al. 2010 [17]	**	**	***	7
Carstensen et al. 2012 [18]	***	**	***	8
Currie et al. 2009 [21]	**	**	**	6
Gu et al. 2013 [22]	***	**	***	8
Onitilo et al. 2013 [25]	**	**	**	6

1 the methodological qualities of the included studies were assessed using the Newcastle-Ottawa scale.

*One point; **two points; ***three points.

**Table 3:** Subgroup analysis of insulin use and CRC incidence with combined RR

	**No. of studies**	**Summary effect**		**Study heterogeneity**	
		**RR**	**95% CI**	**P**	***I***^***2***^
Study design					
Cohort	5	1.25	0.95-1.65	0.002	75.7
Case–control	7	2.15	1.41-3.26	<0.001	89.1
Geographic location					
Europe	3	1.20	0.92-1.57	0.001	85.8
America	5	1.73	1.15-2.60	0.014	67.8
Asia	4	2.55	2.14-3.04	0.373	3.9
Gender					
Male	3	1.02	0.90-1.16	0.512	0.0
Female	3	1.14	0.73-1.77	0.026	72.5

RR, relative risk; CI, confidence interval.

A significant heterogeneity was observed when all the 12 studies were included (I^2^, 91.8%; P < 0.001). However, the heterogeneity was significant in subgroup analyses of cohort (I^2^, 75.7%; P = 0.002) and case–control studies (I^2^, 91.8%; P < 0.001). A sensitivity analysis was conducted after one study which got NOS < 6 stars was excluded and no change of result was observed (RR, 1.65; 95% CI, 1.27 -2.12; I^2^, 87.8%). There was also no change when a fixed-effect method was obtained with all the 12 studies included (RR, 1.35; 95% CI, 1.26 - 1.45; I^2^, 91.8%). No significant publication bias was found in the selected 12 studies (Begg’s funnel plot, symmetrical; Begg’s test, P for bias = 0.086; Eegg’s test, *P* for bias = 0.235).

## Discussion

With the high incidence rate of CRC and very poor prognosis associated with the diagnosis, identification of potential chemopreventive agents is highly desirable. Previous studies showed that aspirin, NSAIDs and statins therapy may be associated with reduced risk of CRC. While insulin is a usually used medicine in the clinic, it is important to detect the effect of insulin use on the risk of CRC. In the present meta-analysis, we observed a significant 69% increased risk of CRC associated with insulin use by combining results from 12 epidemiologic studies, conducted in different populations, which were conducted to investigate the association between insulin use and risk of CRC. Nowadays, much has been known on the role of insulin use in cancer, several systematic reviews and meta-analysis, both on observational and interventional studies, have reported the harmful effect of insulin on cancer.

In our subgroup analyses, there are several interesting results. The subgroup analyses by the study designs showed that cohort studies alone showed no association between insulin use and risk of CRC. Although cohort study would avoid the selection bias and provide better evidence, in this study there were fewer cohorts included and it might be a possible perturbation of the results. Besides, the association of insulin use and risk of CRC in the cohort group was quite closed to the statistical significance level. So it is more reasonable to conclude that insulin use might be a risk of CRC. In the subgroup analyses by the sites, the result in Europe was different with the results from Asia and America. This might be explained by the geographical and ethnic differences. Besides, the environment, genetic factors and dietary factors differences might be possible causes. The stratified analyses by the gender showed that no association between both gender groups was detected. The reason of this outcome was that only 3 studies reported the data stratified by the genders. More well-designed studies are wanted for advanced research.

The potential biological mechanisms that relate insulin and CRC have been studied in several studies. There is evidence suggesting that diabetes is an independent risk factor for colorectal cancer; a meta-analysis showed an overall positive association [29]. Insulin, which is usually used in patients with T2DM, might provide certain role in the increased risk of CRC; concurrently diabetes situation might be associated with the contribution of insulin on the increasing risk of CRC. Insulin and IGF-1 stimulate cellular proliferation; IGF-1 can inhibit apoptosis [28]. Enhanced proliferation of mutated cells or failure to eliminate aberrant cells may contribute to colorectal carcinogenesis. We have previously shown that elevated insulin may contribute to the development of adenomas, the precursors to most colorectal cancer [30].

The strengths of this study include the comprehensive and systematic literature search of observational studies consistency of association between insulin and CRC, ability to evaluate the potential influence of measured confounders on the summary estimates. The likelihood of important selection or publication bias in our meta-analysis is small. During the identification and selection process, we did not exclude any article because of methodological characteristics. With the larger number of studies, we were able to carry out multiple subgroup analyses, evaluate heterogeneity and the presence of publication bias.

We aimed to conduct a rigorous meta-analysis; however, several limitations should be acknowledged. First, several of the included studies enrolled diabetic men from different ethnicities in the same geographic region. Our subgroup analysis was conducted according to region instead of ethnicity. Race has been reported to be one of the strongest risk factors for diabetes mellitus; however, since regional variation in diet is also a confounding factor for DM, we chose to consider location only. Secondly, we did not consider the associations between insulin use and CRC subtypes since there was limited data on this in the included studies, and it prevented classification of the subtypes. Finally, language bias was introduced, as our systematic review since our search included studies written in English only. This decision was based on the difficulty for us to retrieve all available literature in all languages.

## Conclusions

In conclusion, this meta-analysis supports an association between insulin use and CRC risk. To further elucidate this relationship, efforts to quantify insulin dose and duration on the CRC risk at the individual level in large observational studies or randomized trials are needed. These are fundamental steps before suggesting a better and more reasonable insulin strategy to patients with T2DM.

## Competing interests

The authors declare that they have no competing interests.

## Authors’ contributions

SHY conceived the study idea and designed the study. SHY and HB reviewed the literature and performed statistical analyses. SHY and DJ extracted data and drafted the manuscript. SHY, HB and DJ reviewed and edited the manuscript. All authors read and approved the final manuscript.
